# Using the Fuzzy DEMATEL to Determine Environmental Performance: A Case of Printed Circuit Board Industry in Taiwan

**DOI:** 10.1371/journal.pone.0129153

**Published:** 2015-06-08

**Authors:** Sang-Bing Tsai, Min-Fang Chien, Youzhi Xue, Lei Li, Xiaodong Jiang, Quan Chen, Jie Zhou, Lei Wang

**Affiliations:** 1 Management School, Zhongshan Institute, University of Electronic Science and Technology of China, Guangdong, 528402, China; 2 China Academy of Corporate Governance, Nankai University, Tianjin, 300071, China; 3 Law School, Nankai University, Tianjin, 300071, China; 4 Business School, Nankai University, Tianjin, 300071, China; 5 College of Management and Economics, Tianjin University, Tianjin, 300072, China; 6 School of International Business Administration, Shanghai University of Finance & Economics, Shanghai, 200433, China; 7 College of Tourism and Service Management, Nankai University, Tianjin, 300071, China; Southwest University, CHINA

## Abstract

The method by which high-technology product manufacturers balance profits and environmental performance is of crucial concern for governments and enterprises. To examine the environmental performance of manufacturers, the present study applied Fuzzy-DEMATEL model to examine environmental performance of the PCB industry in Taiwan. Fuzzy theory was employed to examine the environmental performance criteria of manufacturers and analyse fuzzy linguistics. The fuzzy-DEMATEL model was then employed to assess the direction and level of interaction between environmental performance criteria. The core environmental performance criteria which were critical for enhancing environmental performance of the PCB industry in Taiwan were identified and presented. The present study revealed that green design (a1), green material procurement (a2), and energy consumption (b3) constitute crucial reason criteria, the core criteria influencing other criteria, and the driving factors for resolving problems.

## Introduction

In recent years, increased international environmental awareness and the prevalence of green consumption concepts have gradually elevated the expectations of the public regarding corporate environmental performance. [1] These enterprises that endeavour to enhance their overall green competitiveness must resolve problems involving green production (GP) and green supply chains (GSCs) [2].

To measure corporate environmental performance, the GP and GSC dimensions of an enterprise must be consolidated, discussed, and compared [3]. The United Nations Environmental Program defined the term green production as “the continuous application of an integrated environmental strategy to processes, products, and services to increase efficiency and reduce hazards to humans and the environment” [4].

GP entails encouraging pollution prevention and emphasising the responsibilities of manufacturers in achieving or maximizing ecological benefits and sustainable development. [3] Green supply chains prompt suppliers to incorporate environmental principles into supply management mechanisms by integrating and collectively considering factors related to product and environmental management. The GSCs are employed to incorporate environmental protection concepts into products and enhance market competitiveness.

Winn and Roome [5] defined corporate environmental performance management as the actions that enterprises adopt in response to the direct and indirect impact that their products, processes, and organisations have on natural ecosystems. Oliver and Abhishek [6] stated that corporate environmental performance management is based on sustainable development and product life cycles. It is defined such management as the incorporation of environmental ideas into enterprise operating philosophies and commitments to internally and externally convey environmental protection concepts.

When implementing corporate environmental performance management, enterprises are able to identify potential impacts that they may exert on the environment and at the same time apply prevention measures. These enterprises integrate environmental objectives and departmental functions in order to implement comprehensive quality control and innovative environmental technologies [7]. This could enhance the corporate image and enable legal penalties to be avoided and to create new business opportunities [4].

In an effective corporate green management system, the entire enterprise emphasises environmental management, complies with environmental regulations, promotes organisation greenification, minimises resource consumption, reduces pollution, and appropriately manages waste [7].

These aspects show how important it is for enterprises to improve their environmental performances, especially those in the PCB industry. The printed circuit board (PCB) industry is a capital-intensive and high-technology industry that requires high levels of energy consumption and emits a great amount of pollution. Its manufacturing process requires large amounts of electricity, water, and chemical solutions, resulting in generation of a substantial amount of waste [8]. The methods by which high-tech companies balance profits and environmental performance are crucial concerns for governments and enterprises.

The PCB industry in Taiwan was used as a case in the present study to examine environmental performance criteria and to determine which ones were critical for improving environmental performance of the PCB industry in Taiwan.

Printed circuit boards (PCBs) are manufactured using numerous high-end technologies such as printing, etching, electroplating, and drilling technologies. Manufacturing PCBs is complex and requires a wide range chemicals, and thus produces gaseous, liquid, and solid wastes. In addition to numerous organic pollutants, the waste generated in the PCB industry includes heavy metals (i.e., copper, lead, and nickel), which are potently toxic to the environment. Without appropriate pollution prevention mechanisms, these wastes inevitably cause severe environmental harm. Therefore, governments and enterprises have consistently emphasised methods for reducing the overall environmental pollution of entire PCB manufacturing chains.

Taiwan is a global leader in PCB manufacturing and is ranked third worldwide in output value. Regarding technology, Taiwan ranks second worldwide, inferior only to Japan, suggesting that the PCB industry in Taiwan has a strong technological advantage. According to the statistics published by the Taiwan Printed Circuit Association, global PCB output in 2012 was US$59.79 billion; of this value, China accounted for US$25.52 billion, representing a global occupancy of 42.7%, followed by Japan (14.4%) and Taiwan (13.4%). The PCBs manufactured in Japan and Taiwan are typically incorporated into high-value products.

The paper is the first, based on prior studies model knowledge, to implement the Fuzzy-DEMATEL model to examine the environmental management performance in the PCB industry in Taiwan. Thus, the contribution of this research is to highlight the environmental performance of the PCB industry in Taiwan by using the Fuzzy-DEMATEL model.

## Literature Review

### Green Production and Green Environment Performance

Green production has 2 primary goals. The first goal is to eliminate or reduce waste generation and environmental pollution, which could harm people and the environment. The second goal is to maximize resource utilization and reduce resource depletion by reusing resources and conserving energy, materials and water [4].

GSC management is applied throughout the manufacturing process, from product design to product recycling. Several dimensions, such as green design, green materials, green suppliers, GP, green sales and packaging, green transport, and green recycling, can be analysed to measure the degree to which GSC management is applied [9].

GSC management is a process for innovating conventional supply chains [9]. Both driving and hindering factors influence the establishment of GSCs [10]. The primary factor driving the establishment of GSCs is that enterprises can seek and form alliances to implement GSC in an intensely competitive market. Within a GSC, enterprises can integrate with upstream and downstream enterprises to combine strengths and increase the benefits received by all members of the supply chain [9].

The primary factor hindering the establishment of GSCs is that, although GSCs can increase the use efficiency of resources and reduce costs to a certain extent, green recycling and waste processing entail considerable costs. These costs may offset benefits and even result in deficits [11].

In GP and GSCs, green design, green material procurement, green manufacturing, green products, green transport, green packaging, and green recycling, are the key strategic factors for corporate green management and constitute the essential criteria for analysing corporate environmental performance [12].

### Environmental Performance Evaluation and Dimensions

To comply with green performance regulations, enterprises develop independent green production management systems by using their extant management systems as a framework, incorporating GP and GSC management into these systems as the problems, and environmental requirements and methods as the tools. These systems enable enterprises to resolve the bottlenecks and difficulties involved in complying with green product requirements and improve the green competitiveness of these enterprises in their respective industries.

Environmental Performance Assessment (EPE) is a systematic procedure. In order to determine the effectiveness of those systems, environmental performance assessment is necessary [13]. The measurement and evaluation involves collecting and evaluating past, present and future data. These data are examined to evaluate enterprises’ management systems, operating systems, and surrounding environmental conditions. Environmental Performance Assessment is based on the concept “no measurement, no management” [14]. EPE entails establishing suitable performance criteria and continually collecting objective and reliable data [15].

Conrad and Morrison [16] asserted that, because of environmental regulations, enterprises may invest their limited resources into unproductive pollution prevention equipment, thereby could reduce their investment in productive equipment. This reduces the productivity of these enterprises and hinders them from considering environmental performance. However, Carter et al. [17] suggested that, by complying with environmental regulations, enterprises can achieve efficient production and improve environmental performance. Tseng and Lin [18] indicated that the goal of mutual collaboration between suppliers and clients in a supply chain is to reduce the negative impact that production exerts on the environment and to maintain environmental performance. Tseng et al. [19] emphasised that enterprises must establish relationships with more appropriate suppliers and engage in strategic procurement to enhance environmental performance. Ahi and Searcy [9] mentioned that several dimensions, namely green design, green material procurement, green manufacturing, green products, green transport, green packaging, and green recycling which significantly influence environmental performance. Zhu et al. [20] indicated that increased environmental performance stimulates the operating performance and increases the market share of enterprises.

**Fig 1:**
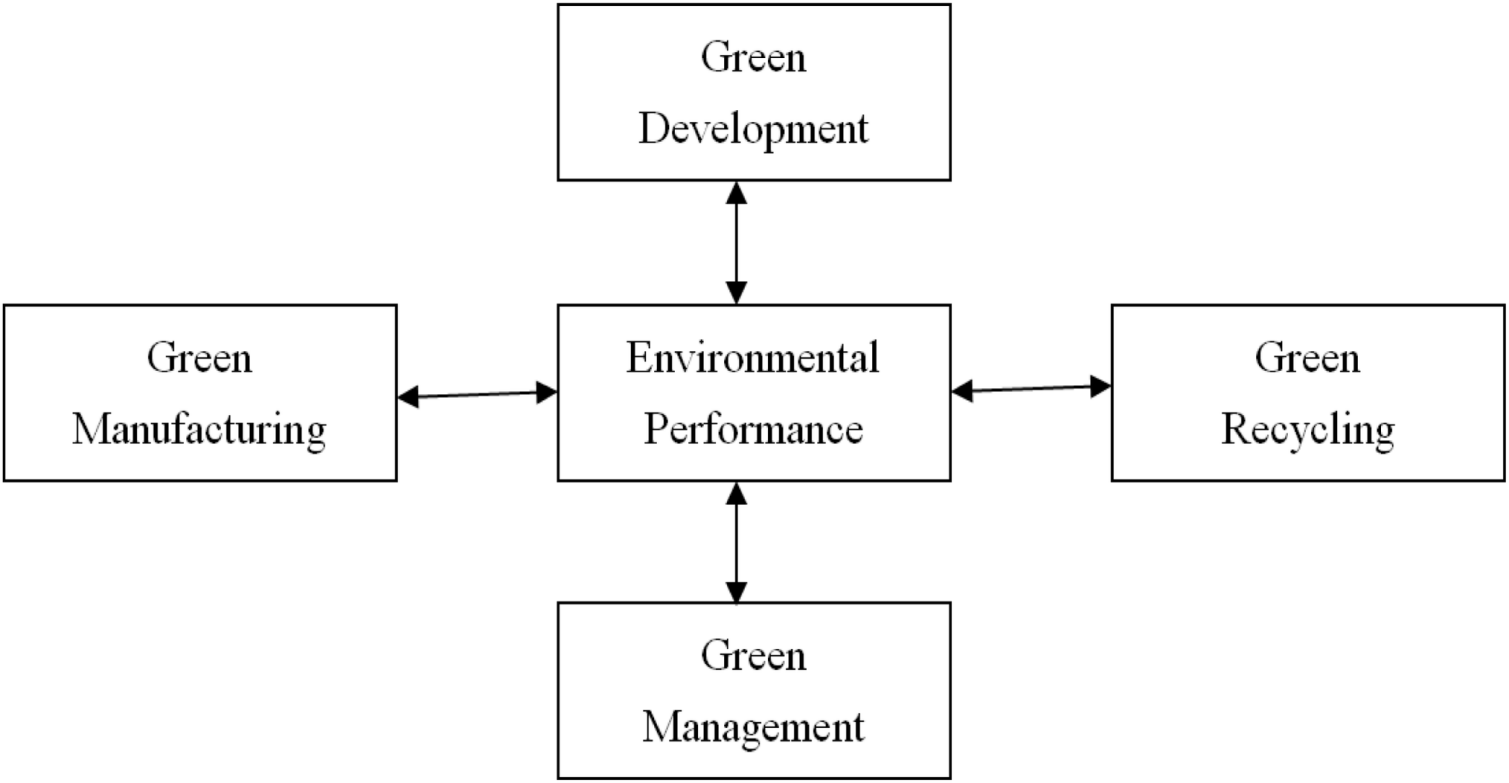
Environmental performance evaluation system.

Based on the aforementioned discussion regarding GP, GSC management and EPE, environmental performance measurement systems were categorised into 4 major dimensions for analysis, specifically, green development, green manufacturing, green management, and green recycling, as shown in Fig 1.

#### Green Development

Green development comprises green design and green procurement. Prior to manufacturing, products are conceptually developed in the planning and design stage. Green products that can be manufactured without violating environmental performance requirements are created through green design. Chu et al. [21] suggested that, in the design of product life cycle processes, green design should be coordinated with procurement to ensure that product and environmental optimisation are interdependently achieved. All components constituting a product must be carefully inspected during the design stage and all suppliers must ensure that toxic substances are eliminated during manufacturing.

Green procurement involves implementing green concepts when selecting and purchasing raw materials from a supplier. In GSCs, suppliers provide green materials to enable manufacturers to improve their environmental performance [22].

#### Green Manufacturing

Green manufacturing refers to enterprises’ attempts to reduce air and water pollution during their manufacturing processes as well as production, waste, pollution and energy consumption. Green manufacturing is the goal that it is minimal impact on the environment, the highest resource efficiency.

#### Green Management

Green management includes green marketing, green corporate image development and green transport. Green marketing emphasises the propagation tools used in conventional marketing, such as promotional activities, advertising, sales representatives, discount sales, public relations and direct marketing. It also involves conveying green messages through products sold to consumers, thereby creating a favourable green corporate image. Tsai and Xue [23] asserted that the primary goal of green marketing is to raise customer awareness towards the influences that environmental deterioration exert on quality of life and, thus, motivating these customers to purchase green products and establish a fundamental understanding towards environmental performance.

The purpose of green transport is to enhance customer satisfaction, achieve centralised distribution, reduce resource consumption and plan effective traffic routes. Green transport entails the demand of manufacturers for green products/raw materials with the supply of those products or raw materials by suppliers and effectively managing delivery services [21].

#### Green Recycling

Green recycling comprises green packaging and product recycling [22]. Typically, consumers discard products once they become unusable. Enterprises can enhance their green performance by guiding consumers to reuse or recycle products through product design and packaging[23]. Green packaging minimises the production of waste once products are discarded, and product recycling promotes the reuse and renewal of products. Both green packaging and product recycling can reduce the burden imposed on the environment.

### DEMATEL Model

The DEMATEL originated from the Natural Sciences and Humanities Research Plan proposed by the Battelle Institute in 1971 [24–25]. During the initial stages of development, the DEMATEL was designed to identify intricate problems in the world such as racism, hunger, environmental protection, and energy conservation. In that period, the DEMATEL was employed in 3 major research fields, specifically, (1) world problem structures; (2) analysing and developing adaptive methods for resolving intricate world problems and (3) reviewing research and methodology data pertaining to world problems [26].

In recent years, the DEMATEL has been employed widely to resolve problems in various fields. Lee et al. [26] examined the effectiveness of the DEMATEL in a technology acceptance model. Wu et al. [27] evaluated the performance criteria of employment service outreach programme personnel. Lee and Hsieh [28] employed the DEMATEL to analyse the causal relationships of service attributes in the telecommunications industry, rearrange the priorities of service attributes, and resolve competitiveness problems. Tsai et al. [29] employed the DEMATEL to examine how manufacturers acquire orders and propose favourable competition strategies.

The framework and computation procedures applied in the DEMATEL consist of the following steps [29]:

**Step 1: Establish measurement scales and determine the direction and degree of influence between factors.** This step entails identifying and defining the various influential factors of complex systems by using data obtained from literature reviews, brainstorming, or expert opinions. In addition, a degree of influence scale is designed to perform pairwise comparisons of the factors and determine the causalities and degree of influence between factors.

**Step 2: Establish a direct relation matrix.** After the significance of the measurement scales is determined, a questionnaire survey method is employed. In this method, experts perform pairwise comparisons of the factors to determine the degree and direction of interactive influence between factors. Consequently, a direct relation matrix is formulated. Each value in this matrix represents the size of an interactive influence between factors. The diagonal values in the matrix are set as 0.

$$X=\left[\begin{array}{cccc} 0 & x_{12} & \cdots & x_{1n}\\ x_{21} & 0 & \cdots & x_{2n}\\ \vdots & \vdots & \ddots & \vdots \\ x_{n1} & x_{n2} & \cdots & 0 \end{array}\right]$$ (1)

Step 3: Calculate a normalised direct relation matrix, where the column vector and maximum value are the thresholds for normalisation.

$$\lambda =\frac{1}{\begin{array}{c} Max\\ 1\leq i\leq n \end{array}\left(\sum_{j=1}^{n}x_{ij}\right)}$$ (2)

N=λX (3)

Step 4: Calculate the direct/indirect relation matrix T, which is also called the total relation matrix.

$$T=\underset{k\to \infty }{\text{lim}}\left(N+N^{2}+\cdots +N^{k}\right)=N{\left(I-N\right)}^{-1}$$ (4)

**Step 5: Calculate the sum of the values in each column and each row.** This step entails summing the values of each column and row in the total relation matrix, where *D*_*i*_ is the sum of the *i* ^th^ row and *R*_*j*_ is the sum of the *j* ^th^ column. The *D*_*i*_ and *R*_*j*_ values represent both the direct and indirect influences between factors.

$$D_{i}=\sum_{j=1}^{n}t_{ij}\begin{array}{cc} & \left(i=1,2,\ldots ,n\right) \end{array}$$ (5)

$$R_{j}=\sum_{i=1}^{n}t_{ij}\begin{array}{cc} & \left(j=1,2,\ldots ,n\right) \end{array}$$ (6)

**Step 6: Illustrate the DEMATEL cause and effect diagram.** In this step, (D + R) is defined as Prominence and *k* = *i* = *j* = 1, 2,….*n*, illustrating the overall influential directions of a service attribute. This value indicates the core level of service attribute *k* in question. The parameter (D − R) is defined as relation, illustrating the difference in the influences of this service attribute. This value indicates the extent of the influence of the service attribute *k* in question; a positive value suggests that the attribute is a cause and a negative value suggests that the attribute is an effect. In the cause and effect diagram, attributes are plotted on the horizontal axis according to the (D + R) value and on the vertical axis according to the (D − R) value. By using images, complex causal relationships are simplified into comprehensible visual structures.

Based on the coordinate positions of (*D*_*k*_ ＋ *R*_*k*_) and (*D*_*k*_ − *R*_*k*_), attributes can be divided into the following 4 types:

- (*D*_*k*_ *—R*_*k*_) is positive and (*D*_*k*_ ＋ *R*_*k*_) is large: This indicates that the attributes are causes, which are also driving factors for solving problems.
- (*D*_*k*_ *—R*_*k*_) is positive and (*D*_*k*_ ＋ *R*_*k*_) is small: This indicates that the attributes are independent and can influence only a few other attributes.
- (*D*_*k*_ *—R*_*k*_) is negative and (*D*_*k*_ ＋ *R*_*k*_) is large: This indicates that the attributes are the core problems that must be solved; however these are effect-type attributes, which cannot be directly improved.
- (*D*_*k*_ *—R*_*k*_) is negative and (*D*_*k*_ ＋ *R*_*k*_) is small: This indicates that the attributes are independent and can be influenced by only a few other attributes.

### Fuzzy Theory

Zadeh, [30] who believed that people’s thought, reasoning, and perceptions of their surroundings are relatively vague, proposed fuzzy set theory. Zadeh [30] experienced difficulty in allocating a precise percentile or number to these concepts because of individuality and subjectivity and, therefore, contended that conventional extremely precise quantification methods cannot be used to resolve people-centred or complex problems completely. The concepts of fuzzy set theory are essential to accounting for the uncertainty and fuzziness of realistic environments. Research subjects are allocated a value between 0 and 1 to indicate their fuzzy degree [31]. People’s subjective judgments are converted into numbers. This conversion compensates the defect of conventional sets in describing events by using binary logic. This method enables research results to comply closely with human thought patterns.

The research objective of fuzzy theory, which was developed based on the fuzzy set, is to recognize the phenomenon of vagueness to handle vague and uncertain situations. Fuzzy theory has been employed and it has shown useful results in various fields, such as artificial intelligence, automatic control, image recognition, medical diagnosis, psychology, decision support, management science, weather forecasting, and environmental assessment [32]. In the context of fuzzy logic, each number between 0 and 1 is regarded as partially correct. By contrast, crisp set concepts dictate that answers are either 1 or 0. Thus, fuzzy logic enables researchers to process fuzzy, ambiguous, and imprecise mathematical judgments. The most commonly used fuzzy numbers are triangular fuzzy numbers, trapezoidal fuzzy number, and Gaussian fuzzy numbers.

The fuzzy linguistic function entails converting linguistic wording into fuzzy numbers and then defuzzifying these fuzzy numbers to obtain explicit values [32–33].

The defuzzification solver employed in the present study uses the smallest and largest fuzzy number to determine the left and right threshold values. The overall integral value is determined based on the weighted average of the membership function. The following 4 steps are subsequently conducted [34–35].

### Step 1: Standardisation.

$$r_{i}^{\text{max}}\;=\text{max}\;r_{j}^{i},\;l_{i}^{\text{min}}=\text{min}\;l_{j}^{i},\;\Delta_{\text{min}}^{\text{max}}=\text{min}l_{ij}$$

Calculate all programmes *a*_*j*_, *j = 1*, *…*, *J*。

$$\begin{array}{l} x_{lj}=(l_{ij}-l_{i}^{\text{min}})/\Delta_{\text{min}}^{\text{max}}\\ x_{mj}=(m_{ij}-l_{i}^{\text{min}})/\Delta_{\text{min}}^{\text{max}}\\ x_{rj}=(r_{ij}-l_{i}^{\text{min}})/\Delta_{\text{min}}^{\text{max}} \end{array}$$ (7)

Step 2: Calculate the left and right standardised thresholds; *j = 1*, *…*, *J*.

$$\begin{array}{l} X_{j}^{ls}=x_{mj}/1+x_{mj}-x_{lj}\\ X_{j}^{rs}=x_{rj}/1+x_{rj}-x_{mj} \end{array}$$ (8)

Step 3: Calculate all explicit values following standardisation; *j = 1*, *…*, *J*.

$$x_{j}^{crisp}=\left[x_{j}^{ls}(1-x_{j}^{ls})+x_{j}^{rs}x_{j}^{rs}\right]/\left[1-x_{j}^{ls}+x_{j}^{rs}\right]$$ (9)

Step 4: Calculate explicit values; *j = 1*, *…*, *J*.

$$f_{ij}=l_{i}^{\text{min}}+x_{j}^{crisp}\Delta_{\text{min}}^{\text{max}}$$ (10)

### Methodology

The major indicators and objectives of environmental performance have been identified through the introduction and the literature review. The present study categorised the environmental performance measurement system into 4 major dimensions and 10 associated criteria, as shown in Table 1.

**Table 1:** The dimensions and criteria of environmental performance.

Dimensions	Criteria
a. Green development	a1 green design
	a2 green material procurement
b. Green manufacturing	b1 air & water pollution
	b2 waste pollution
	b3 energy consumption
c. Green management	c1 green marketing
	c2 green transport
	c3 green image
d. Green recycling	d1 green packaging
	d2 product recycling

### The Questionnaire

The environmental performance fuzzy-DEMATEL questionnaire comprised 4 dimensions and 10 evaluation criteria. The 4 dimensions were green development, green manufacturing, green management, and green recycling. The 10 evaluation criteria were green design (a1), green material procurement (a2), air & water Pollution (b1), waste pollution (b2), energy consumption (b3), green marketing (c1), green transport (c2), green image (c3), green packaging (d1), and product recycling (d2).

The questionnaires were administered between 12 and 30 May 2014. The questionnaires were primarily administered to a group of experts, who provided their personal opinions regarding the environmental performance of the PCB industry in Taiwan. During the survey, the ambiguity of the experts’ subjective judgments was considered. Thus, a linguistic description method was employed to ensure that the evaluation values of the experts’ subjective judgments were expressed properly. Subsequently, each judgment value was expressed as a triangular fuzzy number, which was then placed on a 5-point scale to determine the degree of influence. The influence was ranked as VH, H, L, VL, or NO.

The recipients of the questionnaire were 12 experts, of whom 6 were general managers of PCB enterprises, 3 were academics, and 3 were government officials serving in environmental protection departments. All have more than 15 years of experience in environmental and industrial management. After completion of the questionnaires, the relationships among the 10 criteria of the EPE were assessed, namely, pairwise comparisons of the degree of causal and interactive relationships among the criteria. The researchers of the present study personally visited each expert to explain the content of the questionnaire prior to administration. A total of 12 valid questionnaires were retrieved, yielding an effective recovery rate of 100%.

### The Fuzzy DEMATEL Model

The fuzzy-DEMATEL model combines the fuzzy linguistic aspect of fuzzy theory with the DEMATEL [36]. Applying the DEMATEL in a fuzzy context enables researchers to analyse the causal relationships of fuzzy variables and determine the level of interactive influence between variables.

The computation procedures of the fuzzy-DEMATEL model consist of the following steps: [37–38]

**Step 1: Develop evaluation standards and design a fuzzy linguistic scale.** This step entails substituting conventional measurement scales with a fuzzy linguistic scale to process the ambiguity of human thought. Based on the concepts of Li and Tzeng [38], the present study used triangular fuzzy numbers to determine the degree of interactive influence between variables; (0.0, 0.0, 0.0) numbers denoting no influence (NO), (0, 0.25, 0.5) numbers denoting a very low (VL) influence, (0.25, 0.5, 0.75) numbers denoting a low (L) influence, (0.5, 0.75, 1.0) numbers denoting a high (H) influence, and (0.75, 1.0, 1.0) numbers denoting a very high (VH) influence (Table 2).

**Table 2:** Fuzzy linguistic comparison chart.

Degree of Influence	Score	Triangular Fuzzy Number
Very high influence (VH)	4	(0.75, 1.0, 1.0)
High influence (H)	3	(0.5, 0.75 1.0)
Low influence (L)	2	(0.25, 0.5, 0.75)
Very low influence (VL)	1	(0.0, 0.25, 0.5)
No influence (NO)	0	(0.0, 0.0, 0.0)

**Step 2: Compile expert evaluations.** To evaluate the relationship between the various criteria *C* = {*C*_*i*_|*i* = 1,2,…,*n*}, *p* experts are invited to determine the interactive influences between the criteria by using the fuzzy linguistic scale to conduct pairwise comparisons. Consequently, *p* fuzzy matrices are obtained (${\tilde{Z}}^{\left(1\right)}$, ${\tilde{Z}}^{\left(2\right)}$, …, ${\tilde{Z}}^{\left(p\right)}$). Fuzzy matrix ${\tilde{Z}}^{\left(K\right)}$ is illustrated as follows:

$${\tilde{Z}}^{\left(K\right)}=\left[\begin{array}{l} 0\;{\tilde{z}}_{12}^{\left(k\right)}\cdots {\tilde{z}}_{1n}^{\left(k\right)}\\ {\tilde{z}}_{21}^{\left(k\right)}\;0\;\cdots {\tilde{z}}_{2n}^{\left(k\right)}\\ \vdots \;\vdots \;\ddots \vdots \\ {\tilde{z}}_{n1}^{\left(k\right)}\;{\tilde{z}}_{n2}^{\left(k\right)}\cdots 0 \end{array}\right]\;;\;k=1,2,\cdots ,p$$ (11)

where $z_{ij}^{\left(k\right)}=\left(l_{ij}^{\left(k\right)},m_{ij}^{\left(K\right)},u_{ij}^{\left(k\right)}\right)$, and a triangular fuzzy number (0,0,0) was set for (*i* = 1,2,…, *n*). The term ${\tilde{Z}}^{\left(K\right)}$ represents the initial direct relation fuzzy matrix determined by the *k* ^th^ expert.

**Step 3: Establish a standardised direct relation fuzzy matrix.** Let ${\tilde{a}}_{i}^{\;(k)}$ denote a triangular fuzzy number, where

$${\tilde{a}}_{i}^{\left(k\right)}=\sum_{j=1}^{n}{\tilde{z}}_{ij}^{\left(k\right)}=\left(\sum_{j=1}^{n}l_{ij}^{\left(k\right)},\sum_{j=1}^{n}m_{ij}^{\left(k\right)},\sum_{j=1}^{n}u_{ij}^{\left(k\right)}\right)$$

and

$$r^{\left(k\right)}=\underset{1\leq i\leq n}{\text{max}}\left(\sum_{j=1}^{n}u_{ij}^{\left(k\right)}\right)$$

Subsequently, through a linear scale conversion, the standardised direct relation fuzzy matrix can be expressed as follows:

$${\tilde{X}}^{\left(k\right)}=\left[\begin{array}{l} {\tilde{x}}_{11}^{\left(k\right)}\;{\tilde{x}}_{21}^{\left(k\right)}\;\cdots \;{\tilde{x}}_{1n}^{\left(k\right)}\\ {\tilde{x}}_{21}^{\left(k\right)}\;{\tilde{x}}_{22}^{\left(k\right)}\;\cdots \;{\tilde{x}}_{2n}^{\left(k\right)}\\ \vdots \;\vdots \;\ddots \;\vdots \\ {\tilde{x}}_{n1}^{\left(k\right)}\;{\tilde{x}}_{22}^{\left(k\right)}\;\cdots \;{\tilde{x}}_{nn}^{\left(k\right)} \end{array}\right]\;;\;k=1,2,\cdots ,p$$ (12)

where x˜ij(K)=z˜ij(k)r(k)=(lij(k)r(k),mij(k)r(k),uij(k)r(k)). Based on the basic principles of the DEMATEL, (12) must comply with the assumption that $\sum_{j=1}^{n}u_{ij}^{\left(k\right)}<r^{\left(k\right)}$. Through basic calculation, the average matrix ***X*** can be obtained.

**Step 4: Establish a total relation fuzzy matrix.** To establish the total relation fuzzy matrix **T**, $\underset{w\to \infty }{\text{lim}}{\tilde{X}}^{w}=0$ must first be ensured. The term ${\tilde{X}}^{w}$ represents the triangular fuzzy matrix, which can be expressed as follows:

$${\tilde{X}}^{\left(w\right)}=\left[\begin{array}{l} {\tilde{x}}_{11}^{\left(w\right)}\;{\tilde{x}}_{21}^{\left(w\right)}\;\cdots \;{\tilde{x}}_{1n}^{\left(w\right)}\\ {\tilde{x}}_{21}^{\left(w\right)}\;{\tilde{x}}_{22}^{\left(w\right)}\;\cdots \;{\tilde{x}}_{2n}^{\left(w\right)}\\ \vdots \;\vdots \;\ddots \;\vdots \\ {\tilde{x}}_{n1}^{\left(w\right)}\;{\tilde{x}}_{22}^{\left(w\right)}\;\cdots \;{\tilde{x}}_{nn}^{\left(w\right)} \end{array}\right]\;,\;{\tilde{x}}_{ij}^{\left(w\right)}=\left(l_{ij}^{\left(w\right)},m_{ij}^{\left(w\right)},u_{ij}^{\left(w\right)}\right)$$

Based on the aforementioned Theorem 3.1, the fuzzy matrix can be expanded as follows:

$$\begin{array}{l} \left[l_{ij}^{\left(w\right)}\right]=\left[\begin{array}{l} l_{11}^{\left(w\right)}\;l_{12}^{\left(w\right)}\;\cdots \;l_{1n}^{\left(w\right)}\\ l_{21}^{\left(w\right)}\;l_{22}^{\left(w\right)}\;\cdots \;l_{2n}^{\left(w\right)}\\ \vdots \;\vdots \;\ddots \;\vdots \\ l_{n1}^{\left(w\right)}\;l_{22}^{\left(w\right)}\;\cdots \;l_{nn}^{\left(w\right)} \end{array}\right]\\ \left[m_{ij}^{\left(w\right)}\right]=\left[\begin{array}{l} m_{11}^{\left(w\right)}\;m_{12}^{\left(w\right)}\;\cdots \;m_{1n}^{\left(w\right)}\\ m_{21}^{\left(w\right)}\;m_{22}^{\left(w\right)}\;\cdots \;m_{2n}^{\left(w\right)}\\ \vdots \;\vdots \;\ddots \;\vdots \\ m_{n1}^{\left(w\right)}\;m_{22}^{\left(w\right)}\;\cdots \;m_{nn}^{\left(w\right)} \end{array}\right]\\ \left[u_{ij}^{\left(w\right)}\right]=\left[\begin{array}{l} u_{11}^{\left(w\right)}\;u_{12}^{\left(w\right)}\;\cdots \;u_{1n}^{\left(w\right)}\\ u_{21}^{\left(w\right)}\;u_{22}^{\left(w\right)}\;\cdots \;u_{2n}^{\left(w\right)}\\ \vdots \;\vdots \;\ddots \;\vdots \\ u_{n1}^{\left(w\right)}\;u_{22}^{\left(w\right)}\;\cdots \;u_{nn}^{\left(w\right)} \end{array}\right] \end{array}$$ (13)

The 3 matrices are ordered as follows: .$\left[l_{ij}^{\left(w\right)}\right]=X_{l}^{w},\left[m_{ij}^{\left(w\right)}\right]=X_{m}^{w},\left[u_{ij}^{\left(w\right)}\right]=X_{u}^{w}$

Let $\underset{w\to \infty }{\text{lim}}X^{w}=\text{O}$ and $\underset{w\to \infty }{\text{lim}}\left(\text{I}+X+X^{2}+\cdots +X^{k}\right)={\left(\text{I}-X\right)}^{-1}$, where ***O*** is the zero matrix and ***I*** is the unit matrix.

$$\tilde{T}=\underset{w\to \infty }{\text{lim}}\left(\tilde{X}+{\tilde{X}}^{2}+\cdots +{\tilde{X}}^{k}\right)=\tilde{X}{\left(I-\tilde{X}\right)}^{-1}$$ (14)

Because standardised direct relation fuzzy matrices contain a convergence matrix, the total relation fuzzy matrix ***T*** can be expressed as follows:

$$\tilde{T}=\underset{w\to \infty }{\text{lim}}\left(\tilde{X}+{\tilde{X}}^{2}+\cdots +{\tilde{X}}^{k}\right)=\tilde{X}{\left(I-\tilde{X}\right)}^{-1}$$

Let

$$\tilde{T}=\left[\begin{array}{cccc} {\tilde{t}}_{11} & {\tilde{t}}_{12} & \cdots & {\tilde{t}}_{1n}\\ {\tilde{t}}_{21} & t_{22} & \cdots & {\tilde{t}}_{2n}\\ \vdots & \vdots & \ddots & \vdots \\ t_{n1} & t_{n2} & \cdots & {\tilde{t}}_{nn} \end{array}\right]$$

where, ${\tilde{t}}_{ij}=\left(l_{ij}^{''},m_{ij}^{''},u_{ij}^{''}\right)$. Therefore,

$$\begin{array}{l} Matrix\left[l_{ij}^{''}\right]=X_{l}\times {\left(I-X_{l}\right)}^{-1}\\ Matrix\left[m_{ij}^{''}\right]=X_{m}\times {\left(I-X_{m}\right)}^{-1}\\ Matrix\left[u_{ij}^{''}\right]=X_{u}\times {\left(I-X_{u}\right)}^{-1} \end{array}$$ (15)

Finally, it is to defuzzify fuzzy linguistic values into explicit values.

**Step 5: Illustrate a cause and effect diagram.** The results are then illustrated in a cause and effect diagram to determine the causal relationships and interactive influences between the various criteria.

## Results and Discussion

### Fuzzy DEMATEL Research Results

#### (1)Designing a fuzzy linguistic scale.

The present study used triangular fuzzy numbers to determine the degree of interactive influence between variables: VH, H, L, VL, or NO. (Table 1)**.**

#### (2) Collecting expert opinions.

Twelve experts provided their opinions on the relationships between the various environmental performance criteria. In addition, the experts performed pairwise comparisons to determine the degree of interactive relationships between the criteria. This study used Matlab software to calculate. Based on expert opinions and pairwise comparison results, the directions and degrees of influence between the criteria were determined. The average scores of the expert opinions are listed in Table 3.

**Table 3:** Fuzzy relationships between the various environmental performance criteria.

Criteria	a1	a2	b1	b2	b3	c1	c2	c3	d1	d2
a1	0	VH	L	L	L	H	L	H	VH	VH
a2	L	0	L	H	L	L	L	H	H	H
b1	VL	VL	0	L	H	H	0	VH	VL	0
b2	VL	VL	L	0	L	H	0	VH	VL	0
b3	VL	VL	L	VL	0	H	VL	VH	VL	0
c1	0	0	0	0	0	0	L	H	0	0
c2	0	0	0	0	L	L	0	H	0	0
c3	0	0	0	0	0	H	VL	0	0	0
d1	0	0	VL	VL	VL	VL	L	H	0	H
d2	0	0	VL	VL	VL	VL	L	H	H	0

#### (3) Conversion of fuzzy linguistics.

The fuzzy scale shown in Table 2 was converted into fuzzy numbers (Table 4). In the present study, the degree of influence can be described using 5 linguistic expressions, specifically, VH influence, H influence, L influence, VL influence, and NO influence. By using Eq (1), these expressions can be converted into fuzzy linguistic values, specifically, (0.75, 1.0, 1.0), (0.5, 0.75, 1.0), (0.25, 0.5, 0.75), (0, 0.25, 0.5), and (0, 0, 0), respectively, to establish a direct relation fuzzy matrix (Table 5).

**Table 4:** Converting the fuzzy scale into fuzzy numbers.

Criteria	a1	a2	b1	b2	b3	c1	c2	c3	d1	d2
a1	0	(0.75, 1.0, 1.0)	(0.25, 0.5, 0.75)	(0.25, 0.5, 0.75)	(0.25, 0.5, 0.75)	(0.5, 0.75, 1.0)	(0.25, 0.5, 0.75)	(0.5, 0.75, 1.0)	(0.75, 1.0, 1.0)	(0.75, 1.0, 1.0)
a2	(0.25, 0.5, 0.75)	0	(0.25, 0.5, 0.75)	(0.5, 0.75, 1.0)	(0.25, 0.5, 0.75)	(0.5, 0.75, 1.0)	(0.5, 0.75, 1.0)	(0.5, 0.75, 1.0)	(0.5, 0.75, 1.0)	(0.5, 0.75, 1.0)
b1	(0.0, 0.25, 0.5)	(0.0, 0.25, 0.5)	0	(0.25, 0.5, 0.75)	(0.5, 0.75 1.0)	(0.5, 0.75, 1.0)	0	(0.75, 1.0, 1.0)	(0.75, 1.0, 1.0)	0
b2	(0.0, 0.25, 0.5)	(0.0, 0.25, 0.5)	(0.25, 0.5, 0.75)	0	(0.25, 0.5, 0.75)	(0.5, 0.75, 1.0)	0	(0.75, 1.0, 1.0)	(0.75, 1.0, 1.0)	0
b3	(0.0, 0.25, 0.5)	(0.0, 0.25, 0.5)	(0.25, 0.5, 0.75)	(0.0, 0.25, 0.5)	0	(0.5, 0.75, 1.0)	(0.0, 0.25, 0.5)	(0.75, 1.0, 1.0)	(0.75, 1.0, 1.0)	0
c1	0	0	0	0	0	0	(0.25, 0.5, 0.75)	(0.5, 0.75, 1.0)	0	0
c2	0	0	0	0	(0.25, 0.5, 0.75)	(0.25, 0.5, 0.75)	0	(0.5, 0.75, 1.0)	0	0
c3	0	0	0	0	0	(0.5, 0.75 1.0)	(0.0, 0.25, 0.5)	0	0	0
d1	0	0	(0.0, 0.25, 0.5)	(0.0, 0.25, 0.5)	(0.0, 0.25, 0.5)	(0.0, 0.25, 0.5)	(0.25, 0.5, 0.75)	(0.5, 0.75, 1.0)	0	(0.5, 0.75, 1.0)
d2	0	0	(0.0, 0.25, 0.5)	(0.0, 0.25, 0.5)	(0.0, 0.25, 0.5)	(0.0, 0.25, 0.5)	(0.25, 0.5, 0.75)	(0.5, 0.75, 1.0)	(0.5, 0.75, 1.0)	0

**Table 5:** Fuzzy direct relation matrices.

Criterion	a1			a2			b1			b2			b3			c1			c2			c3			d1			d2		
	x	y	z	x	y	z	x	y	z	x	y	z	x	y	z	x	y	z	x	y	z	x	y	z	x	y	z	x	y	z
a1	0	0	0	0.75	1.0	1.0	0.25	0.5	0.75	0.25	0.5	0.75	0.25	0.5	0.75	0.5	0.75	1.0	0.25	0.5	0.75	0.5	0.75	1.0	0.75	1.0	1.0	0.75	1.0	1.0
a2	0.25	0.5	0.75	0	0	0	0.25	0.5	0.75	0.5	0.75	1.0	0.25	0.5	0.75	0.5	0.75	1.0	0.5	0.75	1.0	0.5	0.75	1.0	0.5	0.75	1.0	0.5	0.75	1.0
b1	0.0	0.25	0.5	0.0	0.25	0.5	0	0	0	0.25	0.5	0.75	0.5	0.75	1.0	0.5	0.75	1.0	0	0	0	0.75	1.0	1.0	0.75	1.0	1.0	0	0	0
b2	0.0	0.25	0.5	0.0	0.25	0.5	0.25	0.5	0.75	0	0	0	0.25	0.5	0.75	0.5	0.75	1.0	0	0	0	0.75	1.0	1.0	0.75	1.0	1.0	0	0	0
b3	0.0	0.25	0.5	0.0	0.25	0.5	0.25	0.5	0.75	0.0	0.25	0.5	0	0	0	0.5	0.75	1.0	0.0	0.25	0.5	0.75	1.0	1.0	0.75	1.0	1.0	0	0	0
c1	0	0	0	0	0	0	0	0	0	0	0	0	0	0	0	0	0	0	0.25	0.5	0.75	0.5	0.75	1.0	0	0	0	0	0	0
c2	0	0	0	0	0	0	0	0	0	0	0	0	0.25	0.5	0.75	0.25	0.5	0.75	0	0	0	0.5	0.75	1.0	0	0	0	0	0	0
c3	0	0	0	0	0	0	0	0	0	0	0	0	0	0	0	0.5	0.75	1.0	0.0	0.25	0.5	0	0	0	0	0	0	0	0	0
d1	0	0	0	0	0	0	0.0	0.25	0.5	0.0	0.25	0.5	0.0	0.25	0.5	0.0	0.25	0.5	0.25	0.5	0.75	0.5	0.75	1.0	0	0	0	0.5	0.75	1.0
d2	0	0	0	0	0	0	0.0	0.25	0.5	0.0	0.25	0.5	0.0	0.25	0.5	0.0	0.25	0.5	0.25	0.5	0.75	0.5	0.75	1.0	0.5	0.75	1.0	0	0	0

#### (4) Calculation of results.

Eqs (12)–(14) were used to establish direct relation fuzzy matrices for *X*_*l*_, *X*_*m*_, and *X*_*n*_. These matrices were then standardised by adopting the *u* column vector in *l* ≤ *m* ≤ *u* and the maximum value of *u* as the baseline for standardisation. Subsequently, the *X*_*l*_, *X*_*m*_, and *X*_*n*_ direct relation fuzzy matrices were determined.

The defuzzification procedures expressed in Eqs (7)–(10) were employed to defuzzify the fuzzy linguistic values and obtain explicit values (Table 6).

**Table 6:** Fuzzy DEMATEL prominence and relation.

Criteria	D_i_			Ri			D_i_ + Ri			Di—Ri		
	x	y	z	x	y	z	x	y	z	x	y	z
a1	0.65	1.25	2.68	0.03	0.22	1.19	0.68	1.47	3.87	-0.54	1.03	2.64
a2	0.49	1.05	2.60	0.10	0.29	1.25	0.59	1.34	3.85	-0.76	0.76	2.50
b1	0.28	0.69	1.93	0.14	0.44	1.56	0.42	1.13	3.49	-1.28	0.26	1.79
b2	0.25	0.65	1.85	0.14	0.43	1.55	0.38	1.07	3.40	-1.30	0.22	1.71
b3	0.21	0.63	1.83	0.21	0.57	1.80	0.42	1.21	3.63	-1.59	0.06	1.62
c1	0.10	0.19	1.14	0.47	1.03	2.56	0.57	1.22	3.70	-2.45	-0.84	0.68
c2	0.14	0.28	1.30	0.19	0.59	1.82	0.33	0.88	3.12	-1.68	-0.31	1.11
c3	0.07	0.15	1.08	0.78	1.41	2.98	0.85	1.56	4.06	-2.91	-1.26	0.30
d1	0.18	0.52	1.71	0.24	0.55	1.68	0.42	1.07	3.39	-1.50	-0.02	1.47
d2	0.18	0.52	1.71	0.24	0.42	1.46	0.42	0.94	3.18	-1.28	0.11	1.47

#### (5) Illustration of the two-theme cause and effect diagram.

**Fig 2:**
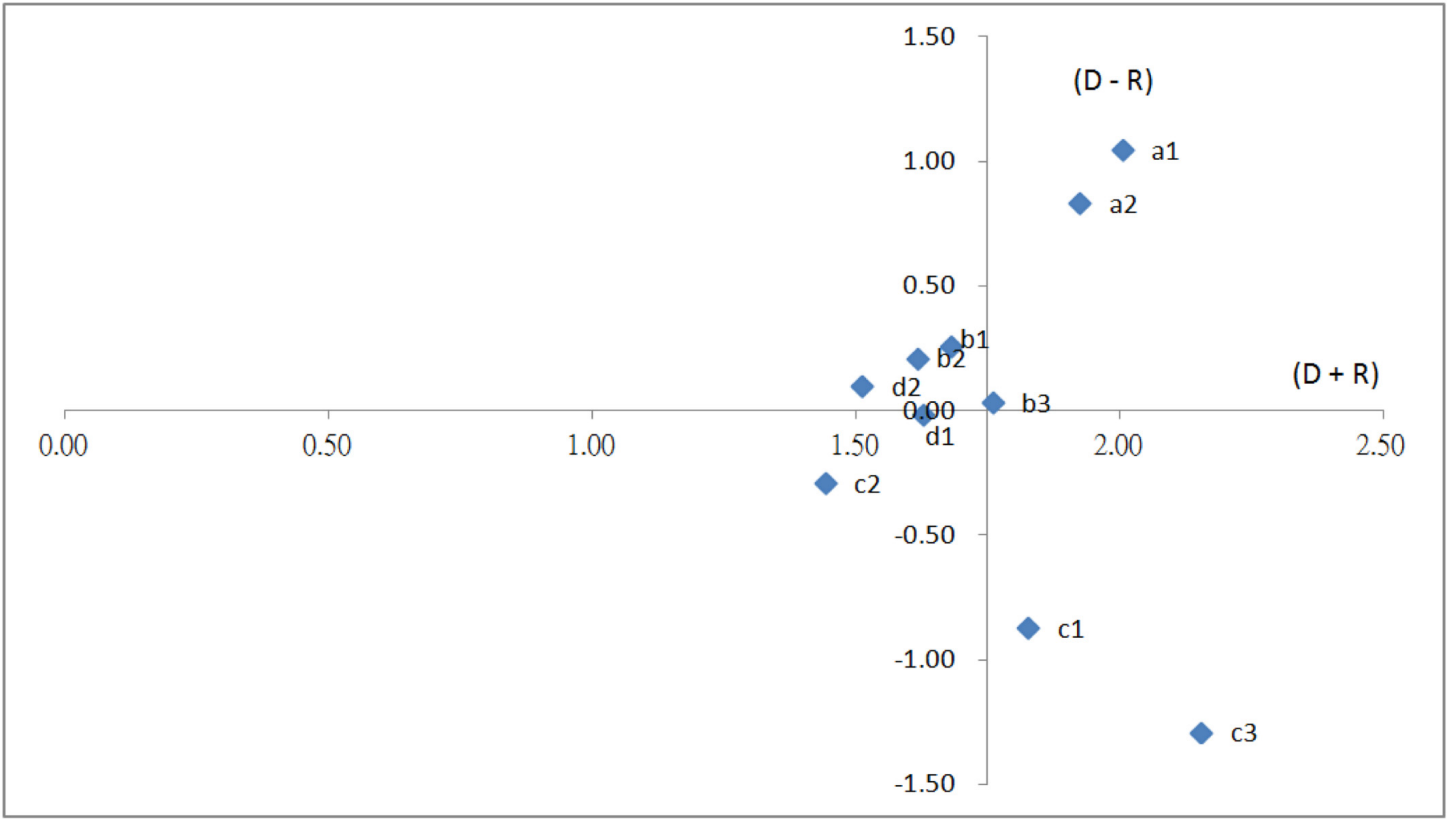
Interactive relationships of the 10 criteria.

Finally, Eq (15) was employed to integrate the total fuzzy relations matrices of *X*_*l*_, *X*_*m*_, and *X*_*n*_ and calculate the (D + R) and (D − R) values (Table 6). The obtained prominence (D + R) def and relation (D − R) def values are listed in Table 7. In addition, the 10 evaluation criteria were plotted on the horizontal axis according to the prominence (D + R) def value and on the vertical axis according to the (D − R) def value (Fig 2).

**Table 7:** Prominence and Relation following defuzzification.

Criteria	(Di + Ri)def	(Di—Ri)def
a1	2.01	1.04
a2	1.93	0.83
b1	1.68	0.26
b2	1.62	0.21
b3	1.75	0.03
c1	1.83	-0.87
c2	1.44	-0.29
c3	2.16	-1.29
d1	1.63	-0.02
d2	1.51	0.10

## Discussion

The 10 criteria were characterised and presented according to relation (D–R) and prominence (D + R), as shown in Table 7 and Fig 2, in between to understand their degrees and directions of interactive influence.

1. *Criteria with high relation and high prominence*: This category comprised green design (a1), green material procurement (a2), and energy consumption (b3). These criteria are characterised as reason criteria, are the core criteria influencing other criteria, and are the driving factors for resolving problems.
2. *Criteria with high relation and low prominence*: This category comprised green marketing (c1) and green image (c3). These criteria influence a minority of the other criteria and the degree of influence is low.
3. *Criteria with low relation and high prominence*: This category comprised air & water Pollution (b1), waste pollution (b2), and product recycling (d2). These criteria are characterised as result criteria, are influenced by other criteria, and cannot be directly improved.
4. *Criteria with low relation and low prominence*: This category comprised green transport (c2) and green packaging (d1). These criteria are influenced by other criteria; however, the degree of influence is extremely low, suggesting that they are relatively independent.

In summary of the aforementioned analyses of the directions and degrees of influence, the present study revealed that green development and green manufacturing are the core dimensions influencing other dimensions and that they are the driving factors for problem solving.

The present study also revealed that enterprises can actively implement the green design (a1) criterion of green development. Enterprises can propose green design management strategies to enhance environmental performance. Additionally, enterprises can implement green material procurement (a2) prior to product manufacturing. Enterprises can incorporate green concepts into the selection and procurement of raw materials from suppliers. Furthermore, enterprises can easily and actively invest in researching the energy consumption (b3) criterion of green manufacturing, thereby enhancing energy efficiency, increasing the use of renewable energy, and reducing resource wastage. By emphasising this criterion, enterprises can achieve the goals of energy conservation and reduction, thereby enhancing environmental performance.

## Conclusion

To measure corporate environmental performance, the GP and GSC dimensions of an enterprise must be consolidated, discussed, and compared. The present study categorised the environmental performance measurement system into 4 major dimensions (i.e., green development, green manufacturing, green management and green recycling), which comprised 10 criteria (i.e., green design, green material procurement, air & water pollution, waste pollution, energy consumption, green marketing, green transport, green image, green packaging and product recycling).

To measure the environmental performance of manufacturers, the present study applied Fuzzy-DEMATEL model to examine environmental performance of the PCB industry in Taiwan. The fuzzy-DEMATEL model combines the fuzzy linguistic aspect of fuzzy theory with the DEMATEL. Applying the DEMATEL in a fuzzy context enables researchers to analyse the causal relationships of fuzzy variables and determine the level of interactive influence between variables. First, fuzzy theory was employed to examine the environmental performance criteria of manufacturers and analyse fuzzy linguistics. The fuzzy-DEMATEL model was then employed to calculate the direction and level of interaction between environmental performance criteria. Finally, the core environmental performance criteria which were critical for enhancing environmental performance of the PCB industry in Taiwan were identified and presented

These environmental performance criteria were green design (a1), green material procurement (a2), and energy consumption (b3) constitute crucial reason criteria, the core criteria influencing other criteria, and the driving factors for resolving problems.
